# P-540. Clinico-Demographic Profile and Outcome of Under-Five Children Admitted with Acute Gastroenteritis in a Tertiary Care Hospital in Nepal: A Descriptive Cross-sectional Study

**DOI:** 10.1093/ofid/ofaf695.755

**Published:** 2026-01-11

**Authors:** Bhishma Pokhrel, Aashis Poudel, Sanjeev Man Bijukchhe, Aashish Giri, Rakesh Pariyar

**Affiliations:** PATAN ACADEMY OF HEALTH SCIENCES, lalitpur, Bagmati, Nepal; Patan Academy of Health Sciences, Lalitpur, Bagmati, Nepal; University of Oxford, Oxford, England, United Kingdom; PATAN ACADEMY OF HEALTH SCIENCES, lalitpur, Bagmati, Nepal; PATAN ACADEMY OF HEALTH SCIENCES, lalitpur, Bagmati, Nepal

## Abstract

**Background:**

Acute gastroenteritis (AGE) is a major cause of morbidity and mortality in children under five, especially in low- and middle-income countries. Despite advances in healthcare, the burden of AGE remains significant in Nepal. This study aimed to evaluate the clinico-demographic profile and management outcomes of under-five children with AGE admitted to Patan Hospital.

**Methods:**

A descriptive cross-sectional study was conducted at Patan Hospital, including 236 under-five children admitted with AGE between January 2021 and December 2023. Data were collected retrospectively from medical records and analyzed using SPSS version 16.

**Results:**

The prevalence of AGE was 14.32% among hospitalized under-five children. The mean age of children was 1.55 years. Most children (77.6%) presented with some dehydration, while severe dehydration was noted in 3.8% of cases. Antibiotics were used in 30.93% of cases, with ceftriaxone being the most commonly prescribed (29%). Zinc supplementation was administered in 38.14% of cases. Intravenous fluids were the primary mode of rehydration in 91.95%.

**Conclusion:**

This study highlighted significant gaps in adherence to clinical guidelines, particularly regarding the underuse of zinc and ORS. The high rate of empirical antibiotic use highlights the need for better diagnostics to guide targeted therapy and reduce antimicrobial resistance.

**Disclosures:**

All Authors: No reported disclosures

